# The effect of a standardized verbal encouragement protocol on peak oxygen uptake during incremental treadmill testing in healthy individuals: A randomized cross‐over trial

**DOI:** 10.1002/ejsc.12044

**Published:** 2024-01-30

**Authors:** Bas Van Hooren, Pleun Van Der Lee, Guy Plasqui, Bart C. Bongers

**Affiliations:** ^1^ Department of Nutrition and Movement Sciences NUTRIM, Institute for Nutrition and Translational Research in Metabolism Maastricht University Maastricht The Netherlands; ^2^ Department of Surgery NUTRIM, Institute for Nutrition and Translational Research in Metabolism Maastricht University Maastricht The Netherlands

**Keywords:** cardiopulmonary exercise testing, cardiorespiratory fitness, maximal heart rate, maximal oxygen uptake, motivation, running

## Abstract

Peak oxygen uptake (V̇O_2peak_) is considered a vital indicator of health and physical fitness that is often measured during incremental exercise testing. While previous research has shown that the attained V̇O_2peak_ during exercise testing can be influenced by verbal encouragement, no or limited details were provided on the verbal encouragement protocol, hereby hampering implementation in clinical practice or research. Moreover, it remains unknown whether motivation modulates the effect of verbal encouragement. This study aimed to develop and examine the influence of a standardized verbal encouragement protocol on the achieved V̇O_2peak_, time to exhaustion (TTE), peak heart rate (HR_peak_), and peak respiratory exchange ratio (RER_peak_) during incremental treadmill testing. As a secondary aim, we investigated whether motivation modulated the effect of verbal encouragement on V̇O_2peak_. 24 healthy volunteers performed two incremental treadmill runs with 1 week in between and received verbal encouragement during only one of the tests. Motivation toward exercise was measured using the behavioral regulation in exercise questionnaire‐2 (BREQ‐2) questionnaire. V̇O_2peak_ (Δ 2.10 mL/kg/min, *p* < 0.001) and RER_peak_ (Δ 2%, *p* = 0.042) were significantly higher with verbal encouragement. In contrast, HR_peak_ (Δ 1.5 beats/min, *p* = 0.225) and TTE (Δ 1.5%, *p* = 0.348) were not significantly different between conditions. Exercise motivation showed a weak and nonsignificant association with the change in V̇O_2peak_ between tests (*r* −0.19, *R* ^2^ 0.037, SEE 2.88, and *p* = 0.367). These findings show that verbal encouragement leads to higher physiological outcomes during incremental treadmill testing, but the magnitude of this effect is not higher for individuals with lower levels of pretest motivation.

## Highlights

- The developed standardized verbal encouragement protocol leads to a higher peak oxygen consumption and respiratory exchange ratio during incremental treadmill testing.
- Peak heart rate and time to exhaustion were not significantly different between tests with and without verbal encouragement, but also showed trends toward higher values.
- Motivation to exercise did not significantly influence the magnitude of the change in peak oxygen uptake between both test conditions.

## 1 INTRODUCTION

The maximum rate of oxygen uptake (V̇O_2max_) during large muscle mass exercise is a vital indicator of an individual's health status and is defined as the maximal oxygen consumption attained during exercise that cannot be increased despite further increases in exercise work rate. Therefore, V̇O_2max_ represents the upper working limit of the cardiorespiratory system. As the plateau phase in oxygen uptake is not always reached, the peak oxygen uptake during exercise (V̇O_2peak_) is often used interchangeably (Day et al., 2003). Nonetheless, participants still need to deliver a maximal effort to ensure valid results. A relatively low V̇O_2peak_ has been associated with numerous adverse health conditions, such as cardiovascular diseases and a higher all‐cause mortality (Ross et al., 2016). Importantly, V̇O_2peak_ has shown to be a more powerful predictor of mortality risk than traditional risk factors, such as hypertension, smoking, obesity, hyperlipidemia, and type 2 diabetes (Ross et al., 2016). As such, V̇O_2peak_ is often assessed in clinical practice and physical exercise training studies as an indicator of cardiorespiratory fitness (Everman et al., 2018; Scott et al., 2018; Wendell et al., 2014). V̇O_2peak_ is also often used to prescribe physical exercise training intensity. For example, training at a percentage of <40%–60% of the V̇O_2_max is often considered to reflect a low (zone 1) training intensity, while training at a percentage of >60%–95% is considered reflective of high training intensity (zone 3) (Iannetta et al., 2020; Seiler, 2010; Tjelta, 2016). Finally, V̇O_2peak_ is also often used in combination with other physiological measures to predict performance in a sports context (Coyle, 1999).

Whether V̇O_2peak_ is being used as an indicator of cardiorespiratory fitness, to prescribe training intensity or to predict performance, accurate assessment of the V̇O_2peak_ is important. While V̇O_2peak_ can be estimated using performance tests (Zwiren et al., 1991) or wearable technology (Düking et al., 2022), incremental exercise testing with respiratory gas analysis is considered the gold standard method. However, the achieved V̇O_2peak_ during such a test can be influenced by factors, such as the modality and duration of the test. Up to 20% higher V̇O_2peak_ values can, for example, be achieved using a treadmill running protocol as compared to a cycling protocol amongst others due to the larger recruited skeletal muscle mass (Marko et al., 2022; Muscat et al., 2015; Rivera‐Brown et al., 1998). With regard to the duration, Yoon et al. (2007) combined their data with those from Astorino et al. (2004) in a regression and showed that an incremental test with a duration of ∼8 min resulted in higher V̇O_2peak_ values among recreational male participants as opposed to tests with both a shorter and longer duration. While this finding is supported by some other studies showing that longer incremental test durations yield lower V̇O_2peak_ values (Buchfuhrer et al., 1983; Jamnick et al., 2018), there are also other studies showing no differences in V̇O_2peak_ between incremental tests of shorter and longer duration (Midgley et al., 2008). Nevertheless, these findings show that the duration of an incremental test can impact the obtained V̇O_2peak_.

Nonexercise‐related factors, such as verbal encouragement, can also influence the obtained V̇O_2peak_ (Andreacci et al., 2002; Dias Neto et al., 2015), although this effect may be modulated by psychological characteristics. A study among highly trained competitive runners, for example, showed that verbal encouragement did not significantly affect the V̇O_2peak_ or the RER as compared to no verbal encouragement, although running time and maximal heart rate were significantly increased (Moffatt et al., 1994). However, for lesser trained individuals, encouragement could be important to reach their maximum potential during exercise testing because untrained individuals may be less intrinsically motivated to provide full effort as compared to trained individuals (Moffatt et al., 1994). In support of this, untrained individuals were shown to reach a higher V̇O_2peak_ with verbal encouragement (Moffatt et al., 1994). Moreover, verbal encouragement during a maximal treadmill test resulted in a 15.7% improvement of exercise time, 8.7% of V̇O_2peak_, and 1.7% of RER in individuals who were classified as more calm and laid back when compared to no encouragement (Chitwood et al., 1997). In contrast, verbal encouragement did not significantly affect exercise time, V̇O_2peak_, or RER in individuals who were more competitively driven (Chitwood et al., 1997). Previous studies investigating the influence of verbal encouragement on exercise performance did not state that they blinded the researchers to the outcomes of the personality characteristics assessment and they did not blind the participants to the study aims, both which may have biased participants to perform better with encouragement.

Although several exercise testing guidelines have recognized the importance of verbal encouragement for achieving a true maximal exercise performance (e.g., American Thoracic Society/American College of Chest Physicians (Ross, 2003), American College of Sports Medicine (ACSM, 2014), ARTP (Pritchard et al., 2021)), the only guidelines that specify what and when something should be said (i.e., by the American Thoracic Society for the 6‐min walk test (ACoPSfCPF, 2002)) are not supported by empirical justification (Midgley et al., 2018) and are not specific to maximal exercise testing. This is likely because the studies that have investigated the effect of verbal encouragement on physiological outcomes during exercise testing have often provided no or limited details regarding the frequency, incentives, and loudness of the encouragement. Therefore, a study that develops and investigates the effect of a standardized verbal encouragement protocol would help in implementing a protocol in clinical guidelines to ensure individuals reach their maximal exercise performance. Therefore, the primary aim of this study was to compare the effect of a standardized verbal encouragement protocol to no verbal encouragement during incremental treadmill exercise testing on the attained V̇O_2peak_ in healthy individuals. Secondary aims were to assess the potential modulating effect of motivation on the effect of verbal encouragement on V̇O_2peak_ and to evaluate the effect of verbal encouragement on time to exhaustion (TTE), heart rate at peak exercise (HR_peak_), and respiratory exchange ratio at peak exercise (RER_peak_). We hypothesized that verbal encouragement would lead to a higher V̇O_2peak_ (and a higher TTE, HR_peak_, and RER_peak_) and that more intrinsically motivated participants would show a smaller increase in V̇O_2peak_ with verbal encouragement.

## 2 MATERIALS AND METHODS

### 2.1 Experimental approach

This randomized cross‐over study consisted of two sessions during which participants performed an incremental treadmill test to voluntary exhaustion. The sessions were separated by approximately 1 week to minimize any influence of fatigue or changes in physical fitness. Participants were randomly assigned to receive verbal encouragement during one of the sessions using an online Research Randomizer and were not informed about the true aim of the study. Instead, participants were informed that they were participating in a test‐retest reliability and validity study. At the second session, participants were also blinded to their result from the first session. The study was conducted according to the principles of the Declaration of Helsinki (Version 2013) and approved by the local ethics committee (FHML‐REC/2021/078).

### 2.2 Participants and sample size calculation

To participate, participants were required to be a healthy trained or untrained male or female aged between 18 and 45 years with a body mass index (BMI) ≤30 kg/m^2^ and to have no injuries at the time of testing. Twenty‐eight participants were eligible to participate in the study and provided informed consent. Participant characteristics are provided in Table 1.

**TABLE 1:** Participant characteristics.

Outcome	Mean ± SD
Age (years)	24.7 ± 5.6
Body mass (kg)	71.5 ± 13.3
Body height (cm)	176 ± 9
BMI (kg/m^2^)	23.6 ± 3.2
Weekly exercise time (min)	216 ± 382
BREQ‐2 score	62.1 ± 13.1

*Note*: Data are presented as mean ± SD.

Abbreviations: BMI, body mass index; BREQ‐2, behavioral regulation in exercise questionnaire‐2; SD, standard deviation.

The sample size was determined a priori using G*Power (version 3.1.9.7) for a dependent *t*‐test based on our primary outcome (i.e., V̇O_2peak_). The alpha level (*α*) and statistical power (1 − *β*) were set at 0.05 and 0.90, respectively. The effect size of 0.711 was based on a comparable study that investigated the effect of verbal encouragement during a multistage fitness test (Dias Neto et al., 2015). This resulted in an estimated sample size of 23 participants. Five additional participants were measured to account for dropout and poor data quality.

### 2.3 Exercise protocol

Participants attended the lab twice for incremental exercise testing on a treadmill (Runrace Treadmill D140, Technogym). A fan was placed in front of the treadmill to maintain a cool body temperature. The incremental protocol started with 5 minutes warming‐up running at 7–9 km/h based on running experience and participant preference. After warming‐up, treadmill speed was increased by 0.5 km/h every 30 s until voluntary exhaustion. When a speed of 20 km/h was reached, exercise intensity was further increased by increasing the inclination of the treadmill by 1° every 30 s until voluntary exhaustion. At exhaustion, the test was ended by the participant either raising their hand or stepping of the treadmill. Total exercise time from start of the warm‐up to termination was recorded. Directly after the exercise test, the 6–20 Borg scale for rating of perceived exertion (RPE) at the moment of test termination was filled out.

### 2.4 Verbal encouragement protocol

A standardized verbal encouragement protocol that integrates the findings from previous research on this topic was used for the test performed with verbal encouragement. In this protocol, the frequency of encouragement increased incrementally as the RER increased with encouragement being provided every 60 s with RER values <1.05, every 20 s with RER values 1.05–1.10, and every 5 seconds to continuously when RER values were >1.10. A minimal frequency of 60 s was used as previous research has shown no effect of verbal encouragement on V̇O_2peak_ with infrequent (once every 3 minutes) encouragement (Andreacci et al., 2002). The volume of the encouragement increased during the test with hand clapping occurring simultaneously with the verbal statements when encouragement was provided continuously. The volume and frequency of encouragement were increased over time to match the increasing levels of fatigue and to reflect previous findings that participants perceive verbal encouragement mostly as helpful during the later stages of a treadmill test (Midgley et al., 2017). An analog‐display sound‐level meter (Sound Meter app, Sound Meter, 2022) was used to ensure that the intensity of the encouragement gradually increased from ∼50 to 70 dB. An increase in loudness was used in an attempt to more strongly motivate individuals with increasing levels of fatigue. This was partly based on previous research showing increasing loudness from 66 to 88 dB lead to increases in maximum voluntary elbow extension force (Johansson et al., 1983). The sound meter was placed on a table located at approximately 1 m distance from the participant and researcher. Prior to the measurements, we determined the motor noise of the treadmill to be <50 dB, thus not interfering with the intensity of the encouragement. The type of incentives used for non‐Dutch participants included: “Very well,” “Way to go,” “You can do it,” “Cheer up,” “You're almost there” “Come on!,” “Good job!,” “Excellent!,” “Come on, push it!,” “Keep it up!,” “Push it!,” and “Let's go”. Some of these phrases have previously been rated as useful during an incremental exercise test (Midgley et al., 2017). Comparable Dutch incentives were used for Dutch participants. Verbal encouragement was given by the same female researcher during the whole study. The researcher was blinded to the motivation of the participants.

In the nonencouragement condition, participants were told that the researchers had to carefully monitor the screen of the CPET system. This was done to avoid the participants from thinking about reasons why the researchers were not providing any encouragement. Further, previous research has shown that reaching a specific performance goal (e.g., a target speed) could lead to task failure in a treadmill running test (Midgley et al., 2017). Therefore, during both tests, we covered the treadmill speed, time, and inclination display using carboard in such a way that participants were prevented from targeting their performance from the first test in the second test, but it still allowed the researcher to see all information. Therefore, during the second session, participants were blinded to the results from their first session.

### 2.5 Outcomes

#### 2.5.1 Questionnaires

Prior to the measurements, all participants were asked to fill out the physical activity readiness questionnaire (PAR‐Q) to check for contraindications to maximal exercise testing. Further, a self‐reported physical activity questionnaire and the behavioral regulation in exercise questionnaire‐2 (BREQ‐2; Markland et al., 2004) were completed to assess habitual physical activity level and the motivation toward exercise, respectively. The BREQ‐2 is a 19‐item questionnaire divided in 5 exercise motivation‐related factors (amotivation, external regulation, introjected regulation, identified regulation, and intrinsic regulation) with a 5‐point scale (0 = not true for me and 4 = very true for me). The following formula was used to score the overall motivation toward exercise: BREQ‐2 score = ∑ ([Amotivation × −3] + [External × −2] + [Introjected × −1]    [Identified × 2] + [Intrinsic × 3]) (Wilson et al., 2012). We also determined the degree of intrinsic motivation from the BREQ‐2 score as detailed in Supporting Information S1. The researcher providing verbal encouragement was blinded to the score on the BREQ‐2 questionnaire.

#### 2.5.2 Anthropometrics

For the anthropometric measurements, a scale (Pino 2.0, SOEHNLE, Backnang, Germany) was used to measure body mass to the nearest 0.1 kg. Participants were weighted immediately before each exercise protocol wearing light clothes and no shoes. A wall‐mounted stadiometer (Seca 206, Seca, Hamburg, Germany) was used to measure standing body height to the nearest 0.1 cm without shoes. BMI was calculated by dividing body mass by the square of body height: BMI = mass/height^2^ (kg/m^2^).

#### 2.5.3 Respiratory gas exchange measurements and heart rate

Participants were instructed to refrain from heavy exercise for 36 h, alcohol for 24 h, caffeine for 6 h, and a heavy meal 2 h prior to the tests. During each running test, participants were breathing through a face mask covering their nose and mouth without detectable air leakage (7600 Series V2, Hans Rudolph Inc) to collect respiratory gasses. An Omnical total‐capture indirect calorimeter (Omnical v6, Maastricht Instruments) was used to measure the rate of oxygen uptake (V̇O_2_) and carbon dioxide production (V̇CO_2_) from which the respiratory exchange ratio (RER) was calculated (V̇CO_2_/V̇O_2_) throughout the tests. Data were gathered with an interval of 5 s. The Omnical was validated with a methanol combustion prior to the test period and was calibrated prior to each test day. Test‐retest reliability of V̇O_2peak_ with this system has previously been shown to be very high (*R* ^2^ 0.97, *p* < 0.001) (Schoffelen et al., 2019). V̇O_2peak_ was calculated as the highest moving average V̇O_2_ value obtained over the last 30 s of the test. V̇O_2peak_ was considered valid and included in the analysis if any of the following criteria was achieved: a RER_peak_ ≥1.10 and/or a HR_peak_ ≥ 95% of the age‐predicted maximum (208 − (0.7 × age)).

Heart rate was recorded using a chest strap (Polar H10) sampling at 1000 Hz. The strap was placed tightly by the researchers on the sternum. An open‐source mobile application (FatMaxxer, https://github.com/IanPeake/FatMaxxer) was used to collect the heart rate data via Bluetooth from which it was exported to Microsoft Excel for further analysis. The highest heart rate obtained in the last 30 consecutive seconds was taken as the HR_peak_.

### 2.6 Statistical analysis

Statistical analysis was performed using open‐source statistical software (JASP version 0.16.2, Amsterdam, The Netherlands). Continuous variables were presented as mean ± standard deviations. Normality of the data was tested using the Shapiro–Wilk test. A paired samples *t*‐test or its nonparametric equivalent (Wilcoxon signed‐rank test) was used to compare V̇O_2peak_, TTE, HR_peak_, and RER_peak_ between the protocols with and without verbal encouragement. Bonferroni corrections were applied for the secondary outcome comparisons (i.e., TTE, HR_peak_, and RER_peak_). A linear regression analysis was performed to assess the influence of motivation on the difference in V̇O_2peak_ between the protocol with and without verbal encouragement with the BREQ‐2 scores as the independent continuous variable and the change in V̇O_2peak_ as dependent variable. The correlation was reported as the Pearson's *r* coefficient (*r*) or Spearman's rho, the coefficient of determination (*R* ^2^), and the standard error of estimate (SEE). Correlations were considered negligible if *r* < 0.1, weak if 0.1 ≥ *r* ≤ 0.39, moderate if 0.40 ≥ *r* ≤ 0.69, strong if 0.70 ≥ *r* ≤ 0.89, and very strong if *r* ≥ 0.90. The significance level *α* was set at 0.05 for all statistical tests.

## 3 RESULTS

Four participants dropped out after the first test due to a knee injury in the period between the tests, fear of treadmill running, illness, and lack of time. Therefore, in total, 24 (19 male and 5 female) participants completed both test days and were included in the data analysis. Ten (42%) participants considered themselves as novice runners and fourteen (58%) as experienced runners. Participant characteristics are reported in Table 1.

**FIGURE 1:**
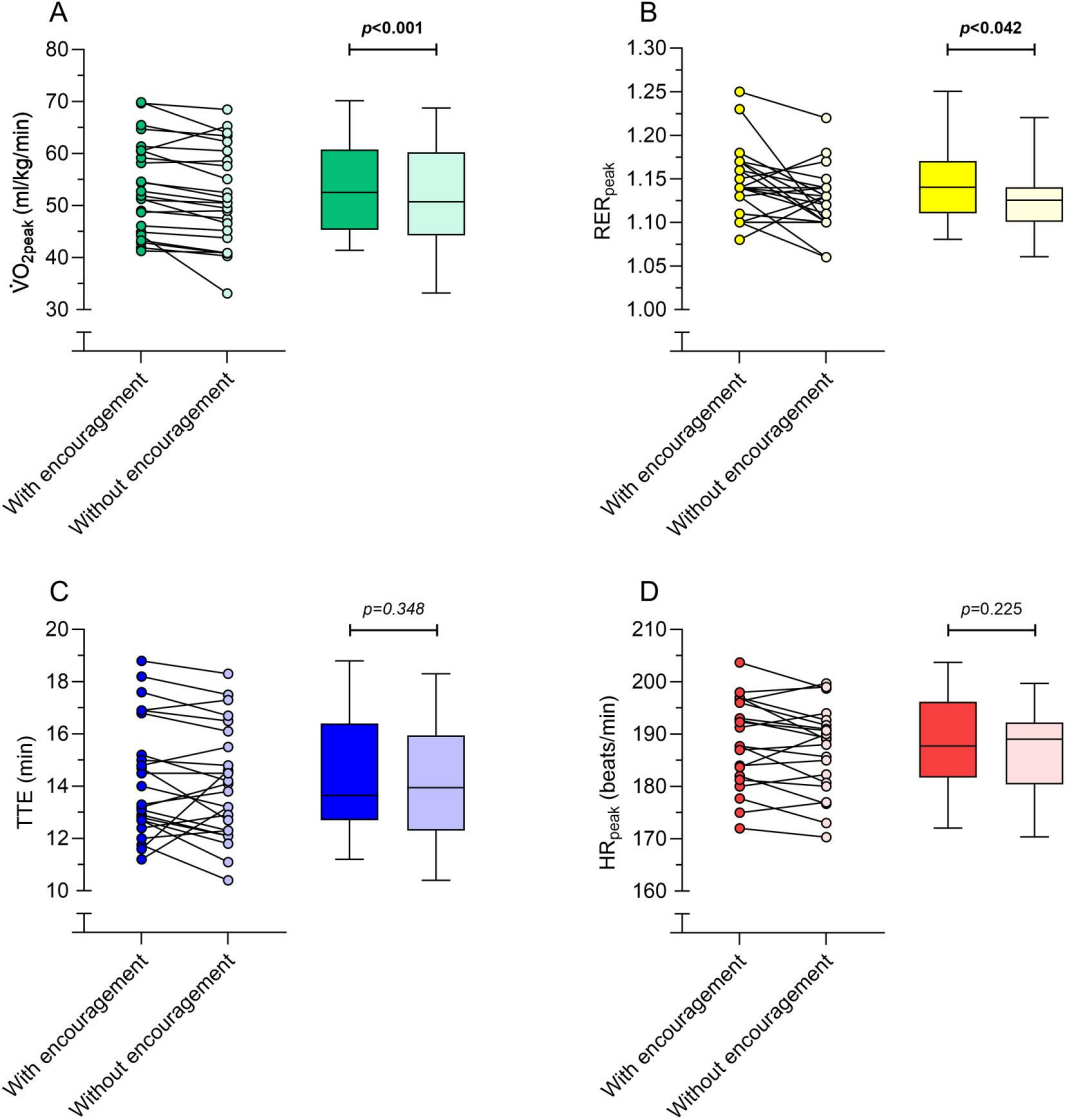
Physiological outcomes during the treadmill exercise tests with and without verbal encouragement. (A) Peak rate of oxygen uptake during exercise normalized for body mass, (B) peak respiratory exchange ratio during exercise, (C) time to exhaustion, and (D) peak heart rate during exercise. Each panel depicts individual data points (left) and box plots (right). For boxplots, the horizontal thick line depicts the median, while the whiskers reflect the minimum and maximum values. Bold *p*‐values indicate significant differences between conditions. HR_peak_, peak heart rate during exercise; RER_peak_, peak respiratory exchange ratio during exercise; TTE, time to exhaustion; V̇O_2peak_, peak rate of oxygen uptake during exercise.

All participants achieved a valid V̇O_2peak_ during both tests based on the previous defined criteria (i.e., RER_peak_ ≥1.10 and/or a HR_peak_ ≥95% of the age‐predicted maximum (208 − (0.7 × age))), therefore, no data were excluded from analyses based on these criteria. The Wilcoxon signed‐rank test showed that V̇O_2peak_ was significantly higher in the test involving verbal encouragement as compared to the test without verbal encouragement (Δ 2.10 mL/kg/min, Δ 4%, *p* < 0.001) (Figure 1A, Table 2). RER_peak_ was also significantly higher in the test with verbal encouragement as compared to without verbal encouragement (Δ 0.022; 2.0%; unadjusted *p* = 0.014, Bonferroni adjusted *p* = 0.042) (Figure 1B, Table 2). Wilcoxon signed‐rank test showed the TTE was not significantly different between conditions (Δ 1.5%, unadjusted *p* = 0.130; Bonferroni adjusted *p* = 0.384) (Figure 1C, Table 2). Among the 24 participants, 21 got their heart rate successfully recorded. For three participants, heart rate was not recorded due to disconnection of the application with the heart rate strap. For the remaining 21 participants, a paired‐samples *t*‐test indicated that HR_peak_ was not significantly different between conditions (Δ 1.5 beats/min, unadjusted *p* = 0.085; Bonferroni adjusted *p* = 0.225) (Figure 1D, Table 2).

**TABLE 2:** Incremental exercise test results with and without receiving verbal encouragement.

	*N*	With encouragement	Without encouragement	*p*‐value
V̇O_2peak_ (ml/min)	24	3957 ± 575	3829 ± 596	**0.010**
V̇O_2peak_ (ml/kg/min)	24	53.7 ± 8.82	51.6 ± 9.39	**<0.001**
RER_peak_	24	1.15 ± 0.04	1.12 ± 0.04	**0.042**
TTE (min)	24	14.3 ± 2.22	14.1 ± 2.15	0.384
HR_peak_ (beats/min)	21	188 ± 8.45	187 ± 8.34	0.225

*Note*: Data are presented as mean ± SD. Significant differences at the 0.05 level are highlighted in bold.

Abbreviations: HR_peak_, peak heart rate during exercise; *N*, number of participants; RER_peak_, peak respiratory exchange ratio during exercise; SD, standard deviation; TTE, time to exhaustion; V̇O_2peak_, peak rate of oxygen uptake during exercise.

**FIGURE 2:**
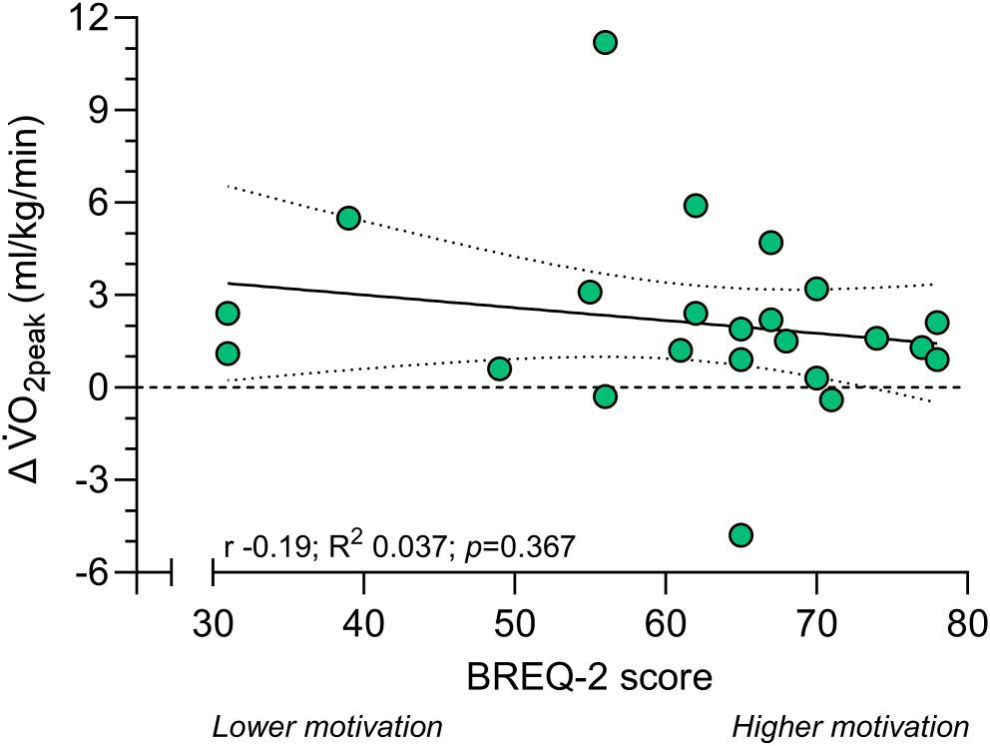
Linear regression analysis of BREQ‐2 scores and the change in V̇O_2peak_ beween incremental exercise tests with and without verbal encouragement. Positive Δ reflects an increase in V̇O_2peak_ when receiving verbal encouragement, and negative Δ reflects a decrease in V̇O_2peak_ when receiving verbal encouragement. BREQ‐2, behavioral regulation in exercise questionnaire‐2; V̇O_2peak_, peak rate of oxygen uptake during exercise.

A weak nonsignificant negative correlation was found between BREQ‐2 scores as the independent continuous variable and the change in V̇O_2peak_ as dependent variable (*r* −0.19, *R* ^2^ 0.037, SEE 2.88, and *p* = 0.367), meaning that a higher motivation was not associated with the change in V̇O_2peak_ between conditions (Figure 2). Similarly, intrinsic motivation was also not significantly associated with the change in V̇O_2peak_ (*R* ^2^ 0.007, *p* = 0.989).

## 4 DISCUSSION

The aim of this study was to assess the influence of a standardized verbal encouragement protocol during incremental treadmill testing on V̇O_2peak_ in healthy individuals. In support of our first hypothesis, we found that the standardized verbal encouragement protocol resulted in a significantly higher V̇O_2peak_ by 4% at the group level as compared to no verbal encouragement. Similarly, we found a significant increase in RER_peak_, but not in HR_peak_ or TTE, although the latter two outcomes also showed a trend toward a higher HR_peak_ and longer TTE with verbal encouragement. Further, in contrast to our second hypothesis, we found no significant and a rather weak correlation between motivation and the change in V̇O_2peak_ between the tests.

The higher V̇O_2peak_ observed in the test with verbal encouragement is in line with previous research among healthy participants that reported significant increases of 8.7%–18% (Chitwood et al., 1997; Dias Neto et al., 2015; Moffatt et al., 1994), but in contrast to a study among highly trained competitive runners that did not report a significant change (Moffatt et al., 1994). However, note that the latter study also reported a percentage improvement of 5%, which is in line with our findings. The different effect magnitudes observed in these studies may reflect different study designs and differences in psychological characteristics of the participants. Specifically, highly trained competitive runners may be (intrinsically) motivated to reach their maximum performance regardless of encouragement, whereas lesser (intrinsically) motivated individuals may perform better with encouragement. Although we found a small trend supporting the hypothesis that individuals with higher overall motivation exhibit a smaller change in V̇O_2peak_ with encouragement, the association was rather weak and nonsignificant (Figure 2). Similarly, we found a nonsignificant trivial association between the change in V̇O_2peak_ and intrinsic motivation. This may partially be due to a rather small variability in overall and intrinsic motivation as it was difficult to recruit highly unmotivated individuals to perform two maximal treadmill running sessions. Alternatively, it could also be argued that overall or intrinsic motivation as assessed using the BREQ‐2 questionnaire may not accurately reflect the willingness to perform maximally in an exercise test, and that other measures, such as personality type (Chitwood et al., 1997), affective attitude toward exercise (Ekkekakis et al., 2021), or conscientiousness (Binboğa et al., 2013), could better reflect this. If so, this could then explain why previous studies showed a difference between type A and B personalities (with type A scoring persons being characterized as competitive, aggressive, impatient, time urgent, and hard‐driving, while type B scorers are patient, easy‐going, tolerant, and relaxed) when receiving verbal encouragement (Chitwood et al., 1997), or in the maximum voluntary contraction between individuals high and low in conscientiousness (Binboğa et al., 2013). However, an important consideration is that previous studies did not state that they blinded the researchers to the outcomes of the personality characteristics assessment, which could have introduced bias by more strongly encouraging individuals that were less motivated. In our study, the researcher was blinded to this outcome. Additionally, previous studies also did not state whether the participants were blinded to the study aim, which may have biased participants to perform better with encouragement. Further, the nonstandardization of verbal encouragement in previous studies makes it difficult to judge whether differences in the frequency, loudness, and incentives used in previous studies could also have contributed to the different effect magnitudes. Indeed, providing verbal encouragement with a frequency of once per 20 or 60 s resulted in a significant greater maximum effort during a treadmill test compared to no encouragement or infrequent encouragement every 180 s in healthy adolescents (Andreacci et al., 2002). Similarly, studies investigating the effect of different incentives on vertical jump or resistance training outcomes have also shown that the exact incentives can differentially affect performance outcomes (Keller et al., 2015; Rendos et al., 2019; Weakley et al., 2020). Finally, the loudness of encouragement can impact force production (Johansson et al., 1983) and may therefore also influence the point of exhaustion. Therefore, in this study, the findings of all prior work were integrated into one verbal encouragement protocol that standardized the frequency, loudness, and incentives. Such protocol may be used in clinical guidelines and future research to improve the reliability and validity of physiological outcomes assessed with incremental exercise testing.

In line with higher V̇O_2peak_, RER_peak_ was also higher in the test with verbal encouragement at the group level. This also indicates that participants were able to achieve a higher level of physiological strain when being verbally encouraged. In contrast, HR_peak_ and TTE did not show significant improvements with encouragement. This may be due to a higher variability in these outcomes as both outcomes also showed a trend toward higher values with encouragement. Improvements in these secondary physiological outcomes with verbal encouragement are also in line with previous research (Chitwood et al., 1997; Dias Neto et al., 2015; Moffatt et al., 1994).

### 4.1 Practical implications

The between‐day reliability in V̇O_2peak_ has been reported to be ∼1–2% or ∼1–1.5 mL/kg/min (Blagrove et al., 2017; Rivera‐Brown et al., 1998). Therefore, the 4% or 2.1 mL/kg/min improvement in V̇O_2peak_ with verbal encouragement in this study is larger than the typical variation of V̇O_2peak_ between different testing sessions. Therefore, we suggest that verbal encouragement should always be provided during an incremental exercise test when the aim is to ensure a *true* maximal performance. Indeed, other studies that do not report significant benefits of verbal encouragement on exercise performance do typically report nonsignificant benefits that collectively reflect a trend toward better performance with verbal encouragement (Bullinger et al., 2012; Moffatt et al., 1994). Achievement of maximal performance is important because the physiological outcomes during a submaximal test may not accurately reflect the health status of an individual or reflect the performance capability when used to predict performance. Moreover, training intensity is often prescribed based on a fixed percentage of V̇O_2peak_ or HR_peak_ (Iannetta et al., 2020), and therefore, an underestimation of these values could result in a training intensity that is lower than may be optimal to improve performance or health.

The employed protocol attempts to standardize the volume of encouragement, the incentives, and the frequency. This standardized nature of the employed protocol may improve the reliability of exercise testing both in a research and clinical practice situation by ensuring more similar encouragement in repeated exercise testing both within the same individual and between individuals. Indeed, in a resistance training setting, verbal encouragement has been shown to improve the reliability of strength assessments (Engel et al., 2019), and a similar effect could be present for physiological outcomes during an incremental exercise test, although this requires further research. Within this context, it is important to emphasize that exact standardization is very difficult to achieve and the proposed protocol may therefore rather assist in ensuring consistency between and within individuals. We believe that the protocol may also be useful for other exercise modalities and incremental tests with a slightly different duration because the encouragement is provided based on physiological responses (i.e., RER) as opposed to a specific time or speed. Finally, it is also of interest that there were different individual responses to verbal encouragement with most individuals performing better on the majority of the outcomes with verbal encouragement and some individuals performing slightly worse (Figure 1). Although this may simply reflect between‐day variability due to, for example, fatigue, it could also reflect positive and negative perceptions of the verbal encouragement (Midgley et al., 2017). However, although some participants mentioned they did perceive the encouragement as distractive, we did not investigate the association between the perception of verbal encouragement and changes in physiological outcomes between conditions, and future studies could explore if the perception of verbal encouragement is associated with the change in physiological outcomes between the tests. Such information could in turn be used to individualize verbal encouragement to maximize exercise performance for each individual.

### 4.2 Limitations

There are also several limitations to this study. First, it was difficult to recruit individuals that were highly unmotivated to perform two incremental treadmill tests, and this may have impacted our ability to establish correlations between motivation and the change in physiological outcomes due to the relatively high homogeneity of the group on motivation. This has previously also been recognized as a potential reason for nonsignificant differences in physiological outcomes during exercise testing between athletes and nonathletes with verbal encouragement (Bullinger et al., 2012). Second, one researcher provided all encouragement, and it is therefore not known how these findings would generalize to different researchers. However, we used a standardized protocol in an attempt to improve the generalizability and hope this study presents a first step toward developing and implementing standardized guidelines into (clinical) exercise testing guidelines. Another limitation is that we included only healthy young individuals. For certain clinical populations, the guidelines may need to be adjusted to provide strong encouragement at lower RER values as these individuals may not reach the RER values used in the present study protocol. Importantly, we intended to develop a standardized protocol that builds upon the findings of previous research, and this may not necessarily represent the optimal verbal encouragement protocol. Future research may therefore further refine this protocol to increase its effectiveness. Additionally, the exact words used in verbal encouragement may influence physiological responses (Hill et al., 2017; Liao et al., 2001; Schücker et al., 2019; Wulf, 2013), and it is, therefore, important to consider the exact words used in particular when translating the protocol to other languages. Finally, we have not investigated the mechanism by which verbal encouragement improved some of the physiological outcomes. However, previous studies have suggested that verbal encouragement may have helped participants to adopt a more dissociative focus instead of a focus on discomfort that in turn could reduce their perceived effort and thereby lead to later task failure and thus higher physiological variables (Andreacci et al., 2002).

## 5 CONCLUSION

In conclusion, findings demonstrate that a standardized verbal encouragement protocol leads to a significant increase in V̇O_2peak_ and RER_peak_, and a small nonsignificant increase in HR_peak_ and TTE when averaged over all participants. Further, a weak and nonsignificant association between motivation and the change in V̇O_2peak_ with verbal encouragement was observed.

## AUTHOR CONTRIBUTIONS

Devising research (Bas Van Hooren, Bart C. Bongers), data collection (Bas Van Hooren, Pleun Van Der Lee), data analysis (Bas Van Hooren, Pleun Van Der Lee), manuscript writing (Bas Van Hooren, Pleun Van Der Lee), editing the manuscript (all authors). All authors provided suggestions, revisions and edits to the manuscript and approved the final version.

## CONFLICT OF INTEREST STATEMENT

The authors declare that they have no conflict of interest. All results are presented clearly, honestly, and without fabrication, falsification, or inappropriate data manipulation.

## Supporting information

Supporting Information S1

Supporting Information S2

## Data Availability

All data generated and used for this publication are available as a supplementary file.
