# Supplementary material for: The effect of a standardized verbal encouragement protocol on peak oxygen uptake during incremental treadmill testing in healthy individuals: A randomized cross‐over trial
Source: Eur J Sport Sci. 2024 Jan 30;24(1):16–25. doi: 10.1002/ejsc.12044 (PMC11235901; doi:10.1002/ejsc.12044)
Supplement: Supplementary file 1 — Supporting Information S1 [file EJSC-24-16-s001.docx]

**Online Supplementary file I Intrinsic motivation determination from the BREQ-2**

The Relative Autonomy Index (RAI) is a composite score that reflects the degree of intrinsic or self-determined motivation towards exercise. The RAI is calculated by multiplying each regulation subscale score by a specific weight, and then summing the weighted scores. The maximum score for the RAI is +20 while the minimum is −24, with higher positive scores indicating higher intrinsic motivation ^1^. Question 1 is for example an externally regulated statement (−2 weighting) “I exercise because people say I should”. A score of e.g. 2 (on a scale of 0–4 of how much you agree with this statement) would be multiplied by the -2 weight, and result in a score of −4 for this question.

**References**

1. Teixeira PJ, Carraca EV, Markland D, et al. Exercise, physical activity, and self-determination theory: a systematic review. *Int J Behav Nutr Phys Act* 2012;9(1):78. doi: 10.1186/1479-5868-9-78 [published Online First: 20120622]
